# The mechanisms of biofilm antibiotic resistance in chronic rhinosinusitis: A review

**DOI:** 10.1097/MD.0000000000032168

**Published:** 2022-12-09

**Authors:** Yanlin Huang, Fengfeng Qin, Sen Li, Ji Yin, Lanxin Hu, Sihan Zheng, Lu He, Hui Xia, Jing Liu, Wenjian Hu

**Affiliations:** Data sharing not applicable to this article as no datasets were generated or analyzed during the current study. a The Affiliated Traditional Chinese Medicine Hospital of Southwest Medical University, Luzhou, China.

**Keywords:** antibiotic resistance, autoinducers, biofilm, chronic rhinosinusitis, quorum sensing

## Abstract

Chronic rhinosinusitis (CRS) is a common but burdensome ailment that is still poorly understood in terms of its pathogenesis. The existence of biofilms on the sinonasal mucosa of individuals with CRS has been proven by current biofilm identification methods. Current treatments for CRS generally include functional endoscopic sinus surgery, biofilm-removing strategies, and limited therapies that target quorum sensing (QS), patients with CRS are often resistant to antimicrobial therapy at degrees achievable by oral or intravenous administration, and even a subset of patients fail to react to either medical or surgical intervention. Multidrug-resistant *Pseudomonas aeruginosa*, *Staphylococcus aureus*, especially methicillin-resistant *S. aureus*, *Streptococcus pneumoniae*, and *Haemophilus influenzae* are the most commonly implicated bacteria in CRS patients, which may lead to the persistence and severity of CRS and antibiotic treatment failure via the formation of biofilms. Resistance to antibiotics is attributed to the 3-dimensional structure and QS of biofilms, and the latter describes the communication of bacteria within biofilms. A better understanding of biofilms in CRS and their contribution to the antibiotic resistance of CRS is critical for novel treatment strategies. This review mainly discusses the special structure of biofilms, QS, and their mechanisms of antibiotic resistance in order to investigate prospective anti-biofilm therapies, suggest future directions for study, and potentially refine the CRS prevention paradigm.

## 1. Introduction

Chronic rhinosinusitis (CRS), one of the most prevalent chronic diseases, affects the quality of life of patients and poses a socioeconomic burden to society.^[1,2]^ Compared with the general population, patients with CRS report a decline in general health and vitality.^[1,3]^ An extensive understanding of how biofilm infection is implicated in CRS is vital, given its severe impact on the quality of life of afflicted individuals and the tremendous cost to the healthcare system.^[4]^ In clinical practice, we have found that a subpopulation of patients diagnosed with CRS (>12 weeks of symptoms and associated findings on computed tomographic scan and nasal endoscopy) remain resistant to cure despite endoscopic sinus surgery and culture-directed long-term antibiotic therapy. Gram-negative bacteria, including *Pseudomonas aeruginosa*, are most commonly associated with this cure-resistant presentation. Therefore, elucidating the various mechanisms underlying biofilm resistance will aid in the development of strategies to control and prevent biofilm formation in disease.^[4]^

The upper airways can condition and clear contaminants from the inspired airstream before they can access the lower respiratory system.^[5]^ Large particulates are eliminated from inhaled air in the anterior naris or nasal vestibule, whereas smaller particulate matter is trapped in a flowing mucus blanket covering the sinonasal mucosa, deeper in the nasal cavity and sinuses. The role of the sinonasal mucociliary system in clearing inhaled particulate matter is an important host defense mechanism. Bacterial colonization, characterized by impaired mucociliary function, may also play a role in the onset or maintenance of the inflammatory process in CRS. In the healthy state, commonly identified bacterial genera in the upper airways include *Staphylococcus*, *Corynebacterium*, *Peptoniphilus* and *Propionibacterium* and so on.^[6]^ Interestingly, the total bacterial load in healthy and diseased sinuses appears to be surprisingly similar across adults. Healthy sinuses are colonized by abundant aerobic and anaerobic bacterial flora, and some of these probiotics may have a protective effect on the organism. However, these microorganisms may also be involved in causing sinus inflammation if the appropriate conditions arise. The lack of host response to normal sinus flora may be critical for the development of sinusitis.^[7]^ Many opportunistic pathogens are found in low abundance in healthy sinuses and, therefore, also have the potential to cause diseases after an acute alteration in the stable baseline microbial community. Based on this hypothesis, CRS could develop through a defined series of dependent events: impaired mucus clearance and disruption of the stable microbiota.^[8]^

There is also a high incidence of CRS in children in clinical settings and like adults with CRS, pediatric CRS patients are strongly affected by biofilms.^[9]^ Studies have shown that the rate of biofilm positivity is much higher in the sinuses of children with CRS than in healthy children or children with acute rhinosinusitis.^[10]^ In addition, studies have also found mature biofilms in 95% of adenoids excised from patients with CRS, suggesting a strong association between the development of CRS and adenoid biofilms.^[11]^ In exception to the paranasal sinuses, the presence of biofilm in other parts of the body can cause corresponding refractory infections such as endocarditis, osteomyelitis, urinary tract infections, chronic prostatitis, periodontitis, chronic lung infections, middle ear infections, and various other infections.^[12]^ Furthermore, some studies have shown that bacterial biofilms can exist on the surfaces of some medical devices implanted in the body like joint prostheses, breast implants, heart valves, and defibrillators, and become the culprit of post-implant infections.^[13]^

## 2. Biofilms in CRS

### 2.1. Definition and formation of biofilm in CRS

Biofilms are commonly described as communities of microorganisms that adhere to a surface and are embedded in a protective, self-produced extracellular matrix (ECM).^[14]^ This matrix is composed of a mixture of biopolymers, primarily polysaccharides, but also contains proteins, lipids, and nucleic acids. These biofilm components facilitate cell coherence and cell surface attachment.^[4]^ Biofilms are natural states in which bacteria prefer to exist, and provide a mechanism for increased survival. Since the ECM protects bacteria against antibodies, phagocytosis, antibiotic penetration, and complement binding, bacteria in a biofilm community are up to 1000 times more resistant to antibiotic therapy.^[15]^

**Figure 1.**
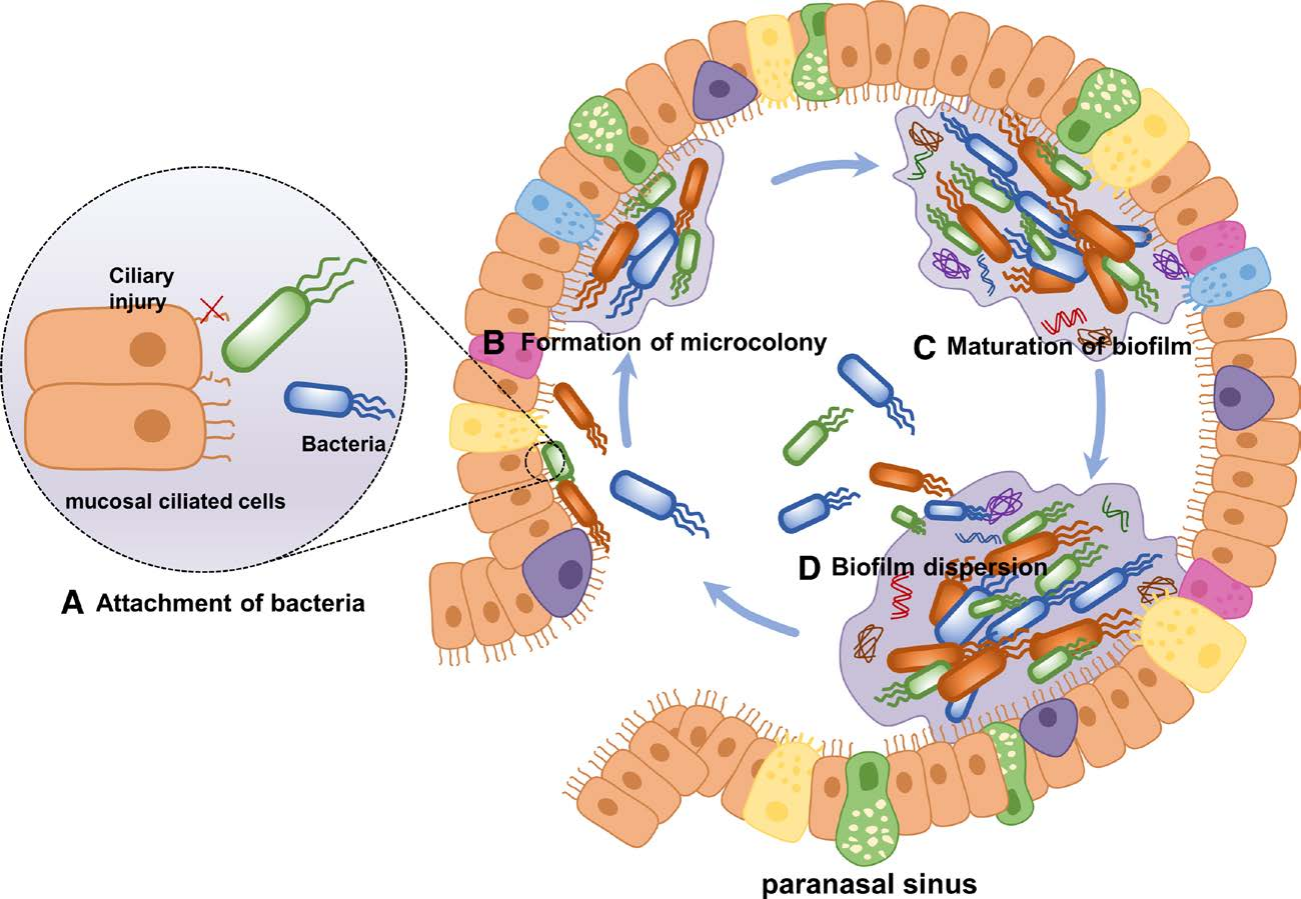
Four stages in biofilm formation. (A) Bacteria attach themselves to a surface. (B) Bacteria generate EPS by surrounding themselves and then forming a microcolony. (C) With the reproduction of bacteria and increasing of EPS, the biofilm gradually mature. (D) Mature biofilms release bacteria, allowing them be planktonic. EPS = extracellular polymeric substances.

Biofilm formation comprises 4 major episodes, beginning with cellular adhesins and progressing to the formation of microcolonies, biofilm maturity, and ultimately biofilm dispersion.^[12,16]^ The 4 steps are illustrated in Figure 1. The first step in biofilm formation by bacteria is to attach to a surface.^[17]^ The surface proteins of bacterial cells assist in cell adherence to the surface, resulting in irreversible attachment.^[18]^ Generally, a crucial host defense mechanism that clears inhaled particulate matter is sinonasal mucociliary function, which relies on antimicrobial peptides in nasal secretions. Antimicrobial peptides found in the sinonasal mucosa are components of the innate immune system and are capable of protecting mucosal surfaces from acute microbial infections caused by bacteria with a planktonic phenotype. However, antimicrobial peptide expression is downregulated in people with innate immune deficiencies, increasing bacterial attachment sites on the mucosal cell membrane and increasing the likelihood of biofilm formation.^[19]^ After bacterial cells become irreversibly attached to a surface, they generally begin to proliferate and generate extracellular polymeric substances (EPS).^[14]^ The generation of EPS culminates in the construction of a biofilm matrix that serves as a shelter for all bacteria attached to this limiting area.^[20]^ Bacterial division combined with constant EPS generation results in the construction of an early biofilm that matures over time and eventually becomes a 3-dimensional (3-D) structure. They contribute to this 3-dimensional (3-D) structure, and are also important for preserving it.^[21]^ After maturation, biofilms undergo a process known as dispersion. At this stage, the persistent existence of bacterial biofilms in the body makes it a bacterial hatchery, causing some bacterial cells to leave the biofilm and revert to their planktonic state, resulting in repeated mucosal inflammation.^[22]^ Meanwhile, some studies have suggested that these bacteria remain continuously colonizing and unleashing, causing unremitting damage to the mucociliary epithelium and thus destroying the function of the normal nasal mucociliary transport system.^[23]^ Mucous stasis within the sinus cavity predisposes to further biofilm formation.^[24]^

### 2.2. Mixed biofilms in CRS

The existence of biofilms in CRS was initially observed in patients with chronic sinusitis in 2004 using scanning electron microscopy.^[25]^ Following the discovery of biofilms in CRS, a few studies have attempted to pinpoint the bacteria responsible. A complex polymicrobial community of both bacteria and fungi exists within biofilms in CRS. Many types of bacteria have been identified in CRS, including *Staphylococcus aureus*, *P. aeruginosa*, *Streptococcus pneumonia*e, *Haemophilus influenza*e, and *Moraxella catarrhalis*. Among these, *S. aureus* biofilms have been most commonly implicated in recalcitrant CRS.^[15]^ In 1 study, *S. aureus* was found in 50% of biofilms from CRS patients, whereas *P. aeruginosa* and *H. influenza*e were found in 22% and 28% of the cultures, respectively. Other bacterial species that can form biofilms in patients with CRS include *Streptococcus viridans*, *coagulase-negative staphylococci*, *Enterococcus faecalis*, and *S. viridans*. Some anaerobes, including *Propionibacterium* and *Corynebacterium*, are also involved in CRS.^[15]^ In this milieu, interactions between organisms are of significant clinical interest. In a study performed by Peters et al, *S. aureus* physically interacted with the hyphae of *Candida* when grown together in vitro to create a dual-species biofilm.^[26]^ Interestingly, not only may the connection between *S. aureus* and fungal hyphae provide a route for epithelial penetration, but also the species interaction may result in a shift in *S. aureus* virulence factor expression. Therefore, this association may promote a clinical *S. aureus* infection in CRS by elevating its virulence via an unclear mechanism.^[27]^ Fungal components, in addition to bacterial components, have also been hypothesized to play a role in biofilm development.^[28]^ Given some in vitro results, these findings support the idea that fungal species play a role in the pathophysiology of CRS and may facilitate *S. aureus* infection. The production of superantigen toxin, which causes nonspecific T-cell activation, is 1 possible mechanism by which *S. aureus* causes inflammation in CRS. However, the effect of organism interactions with fungal species on the development of *S. aureus* superantigens is unknown. Similarly, the effects of this interaction on the cytokine profile and clinical signs of the condition remain to be confirmed.^[4]^

## 3. Factors contributing to antibiotic resistance in CRS

### 3.1. Structural factors

The capacity of biofilms to resist antibiotics is determined by their structure, in which EPS boosts the organism’s adherence and colonization of a surface.^[16]^ In addition to this structural factor, bacteria develop biofilms when they detect changes in the environment in which they live. As a result of the stressful environment, bacteria respond by building a biofilm to protect themselves.^[29]^

#### 3.1.1. EPS: a physical barrier stopping antibiotics in paranasal sinus killing bacteria in biofilm.

The ECM of microorganisms is the most studied biofilm element and plays a critical role in biofilm antibiotic resistance.^[16,30]^ Polymeric compounds, which vary in structure and content among microorganisms, are found in this matrix.^[16]^ Organic polymers present in EPS include polysaccharides, lipids, rare sugars, humic acids, proteins, carbohydrates, and extracellular deoxyribonucleic acid (DNA).^[16,31]^ The self-produced, energy-demanding process of EPS synthesis is attributed to specific environmental conditions.^[32]^ EPS further affects the density, water content, charge, and mechanical stability of biofilm cells. Flemming et al metaphorically referred to EPS as “the house of biofilm cells.” EPS-oriented changes to the aforementioned features determine the lifestyle of bacteria in biofilms in the local environment. This is due to the fact that biopolymers in EPS are hydrated to create a matrix that keeps the cells together and allows for surface adherence, as well as their sorption capabilities, which allow the biofilm organisms to absorb nutrients from the environment.^[20]^ The primary function of EPS is to serve as an impermeable barrier to antimicrobials and stress factors.^[16,32]^ Besides, the matrix structure generates nutritional gradients that, together with cell signals, result in the emergence of persister cells and spores, which are highly resistant subpopulations supervising the antibiotic resistance mechanism.^[33]^

#### 3.1.2. General stress response (GSR): a low metabolic state existing in bacteria inside the CRS biofilm.

With the reproduction and division of bacteria, the accumulation of waste products or oxygen and nutrient depletion within a biofilm can activate the general stress response (GSR), a low metabolic state in which bacteria can exist indefinitely.^[4]^ In CRS biofilms, GSR boosted the emergence of a heterogeneous colony of slow-growing persister cells. Persister cells, in contrast to antibiotic-susceptible cells in the surface layers of biofilms, are exceedingly antibiotic-tolerant and occupy the innermost core.^[34]^ These persister cells, which lie in a dormant state, likely play a role in the recalcitrance of biofilm-mediated illnesses.^[35]^ Persister cells that are less sensitive to antibiotics are capable of resisting high levels of antibiotics and aggravate infection by relocating to other host sites and later forming new biofilms possessing the original population’s resistant phenotype.^[35,36]^

Under low-oxygen conditions, cellular respiration is impaired, which in turn activates programmed cell death in bacteria.^[37]^ In this way, the bacterial cells dissolve and release specific proteins and extracellular DNA (some components of the EPS) into the surroundings, which increases the strength of biofilms.^[16,37,38]^ Similarly, Cramton et al discovered that polysaccharide intercellular adhesin (PIA) was also generated at higher levels when bacteria were exposed to lower oxygen concentrations.^[39]^ Polysaccharide intercellular adhesin (PIA), a polymer produced by *S. aureus*, is used as cellular adhesin. Growing the generation of the aforementioned polymer improves cell adhesion, a critical step in biofilm formation.^[16]^ Therefore, in the GSR state, bacteria are less sensitive to antibiotics and much less susceptible to growth-dependent antimicrobial killing.^[4]^ Bacteria in the deeper layers of biofilms survive antibiotic treatment owing to this unique mechanism of resistance. After antibiotic treatment is stopped, the nutrients released by dead bacteria can provide energy support for surviving bacteria, resulting in the rapid proliferation of bacteria. In just a few hours, the original state of biofilm flora can be restored. Biofilm infection generally stops only if the surfaces of the bacterial colonies can be surgically removed entirely. However, because the naked eye cannot detect the exact position and degree of mucosal biofilm adhesion during endoscopic sinus surgery, the aim of completely removing biofilms is not easily achieved.^[23]^

### 3.2. Quorum sensing (QS)

W.C. Fuqua coined the term “quorum sensing” to define the cell-cell communication of bacteria.^[40]^ His discoveries were based on studies by Tomasz and Nealson, who discovered autoinducer activity in *Vibrio fischeri* as early as 1965 and 1970, respectively.^[16]^ Quorum sensing (QS) plays a crucial role in microbial colonies because bacteria can collectively gather information about their community density, coordinate community behaviors, and implement gene expression in response to fluctuations in the surrounding community concentrations.^[16,41]^

#### 3.2.1. Autoinducers (AIs): signaling molecules of cell-to-cell communication.

Autoinducers (AIs) are extracellular signaling molecules that regulate cell communication through secretion, detection, and responses.^[42]^ The concentration of AIs may be monitored by the population density of bacteria in the environment, and is said to be directly related to the density of the bacterial community, since an increase in the bacterial population leads to an increase in the AIs concentration.^[16,37]^ The principles of the QS system in biofilms rely particularly on the release of AIs by bacteria in a given community. Two QS systems in *Staphylococcus*, the accessory gene regulator (agr) system and LuxS/AI-2 system, have been identified. The agr system relies on communication among different species of bacteria as autoinducing peptides (AIPs), which are the signaling molecules of QS. The LuxS/AI-2 system generates AI-2 molecules that function in both gram-positive and gram-negative communication.^[43]^

#### 3.2.2. Agr system of QS.

As mentioned above, EPS consists of a large number of organic polymers, such as polysaccharides, rare sugars, humic acids, proteins, carbohydrates, lipids, and extracellular DNA, which are generally encoded by accessory genes, enabling the organism to elude host defenses, cling to cells and the tissue matrix, moving throughout the host, and breaking down cells and tissues for both nutrition and defense against host defenses.^[16,44]^ Gram-positive bacterial agr loci contain genes encoding 4 proteins, AgrD, AgrB, AgrC, and AgrA, which can regulate Agr-mediated gene expression of virulence factors and promote cellular processes, such as sporulation.^[45,46]^

**Figure 2.**
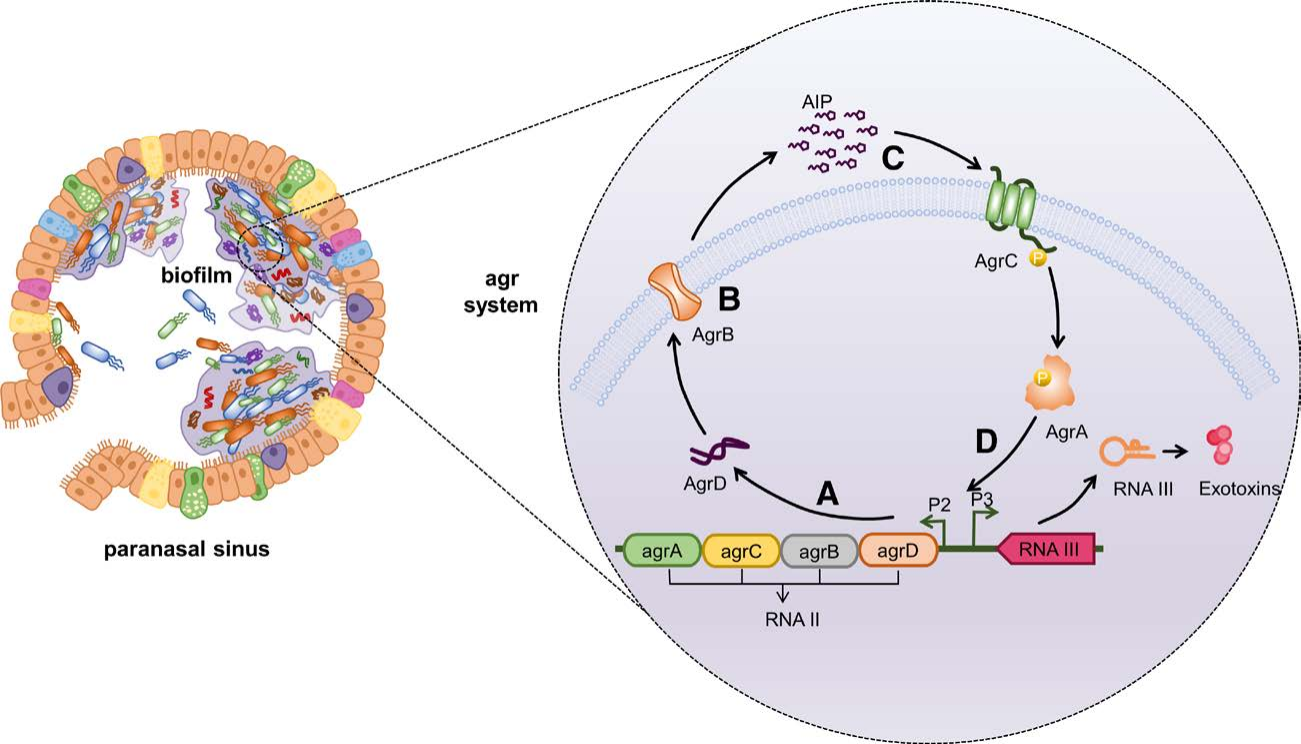
The *Staphylococcus aureus* agr QS system. The agr locus contains 4 genes: agrB, agrD, agrC, and agrA, which encode a transmembrane protease, a peptide, a histidine kinase, and a response regulator, respectively. (A) The agrD gene codes for a prepropeptide AgrD. (B) The AgrD is processed by and secreted through AgrB, the product of the agrB gene. (C) Mature AIPs are secreted to the extracellular milieu to bind to and activate the membrane-bound AgrC. (D) The response regulator AgrA gene is phosphorylated, which promotes transcription of the RNA III. Agr = accessory gene regulator, AIPs = autoinducing peptides, QS = quorum sensing, RNA = ribonucleic acid.

The autoinducer of the *S. aureus* agr system is a self-induced peptide called AIP, whose biosynthesis requires the peptide precursor AgrD and integral membrane endopeptidase AgrB.^[44]^ The gene product of AgrB is a transmembrane endopeptidase that mediates the truncation, cyclization, and secretion of AIP, and the mature AIP is then transported out of the cell via AgrB. There are 4 groups of *S. aureus* AIP that are encoded by a specific AIP peptide sequence.^[45]^ Mature AIPs are 7 to 9 residues in length, with a 5-membered ring formed between the C-terminus via a thiolactone bond and a sulfur atom from a central cysteine.^[47]^ Positive signaling communication only occurs between a specific AIP and its cognate AgrC transmembrane domain as AIPs and their cognate receptors coevolve.^[47]^ First, AgrA is activated by transferring the phosphate group of active AgrC to an aspartate residue on the AgrA response regulator, and then activated AgrA attaches to the promoter region of target genes, utilizing its DNA-binding domain to control or suppress their expression.^[45,48]^ In the well-studied *Staphylococcus cerevisiae* system, AgrA induces the expression of regulatory ribonucleic acid (RNA) III, which subsequently mediates many of the critical transcriptional changes that occur when the gr system is triggered.^[16,49]^ This process is illustrated in Figure 2.

The role of the agr system can be generally summarized in 2 aspects: downregulation of adhesion production and upregulation of δ-hemolysin and protease generation. Adhesion is of great importance in the early steps of biofilm formation, while δ-hemolysin has a negative impact on the maturation of the biofilm because of its detergent characteristics.^[50]^ Proteases can diminish biofilms by degrading required proteins such as adhesins and agglutinins.^[44]^ Despite the fact that agr expression increases biofilm mass sufficiently, its effects are confounded topically: agr is activated very locally during biofilm formation, and clumps of agr-expressing organisms quickly separate from each other, leaving a hole in the film that is quickly filled by new, agr-expressing organisms growing in from the periphery.^[44,51]^ Agr expression is dramatically reduced as the biofilm matures, and many bacteria lose viability.^[44]^

#### 3.2.3. Luxs/ai-2 system of QS.

**Figure 3.**
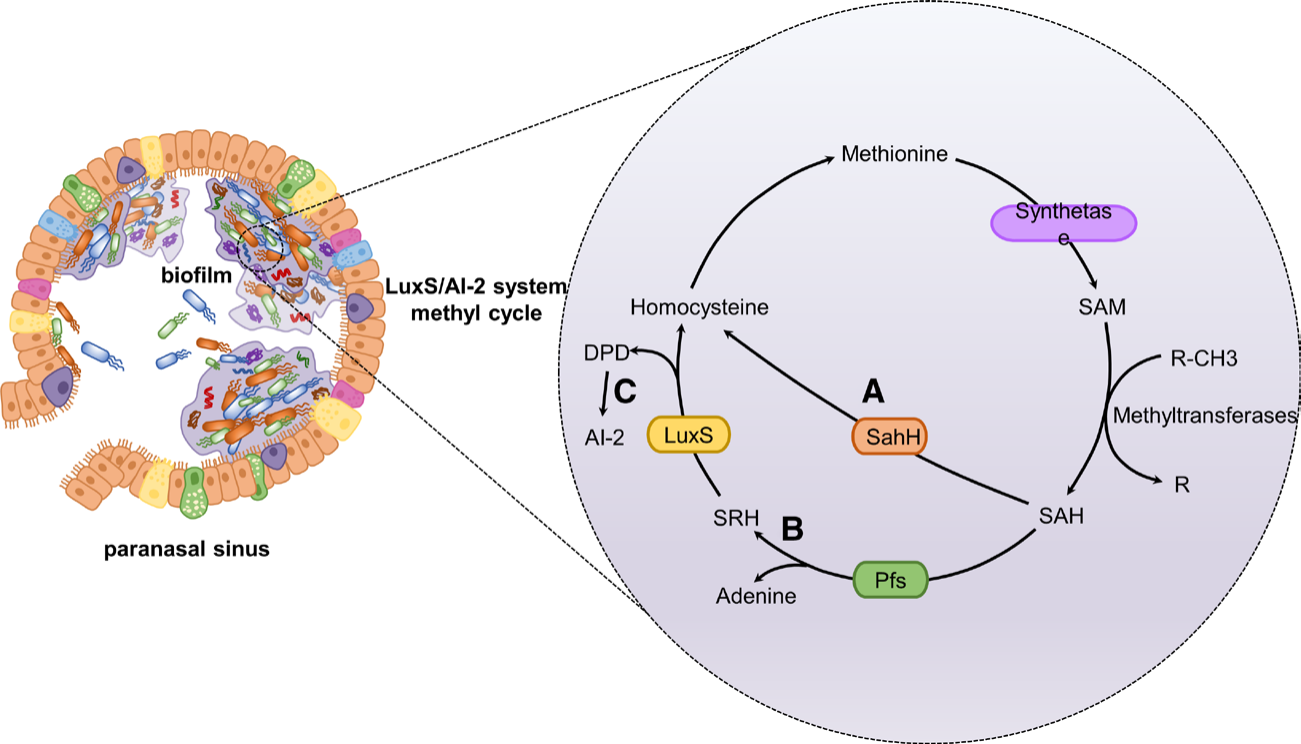
The LuxS/AI-2 system, the active methyl cycle. (A) A 1-step reaction using the enzyme S-adenosyl homocysteine (SAH) hydrolase (SahH). (B) A 2-step reaction: The conversion of S-adenosyl homocysteine (SAH) to adenine and S-ribosylhomocysteine (SRH) is catalyzed by S-adenosylhomocysteine nucleosidase (Pfs). The conversion of S-ribosylhomocysteine (SRH) to homocysteine and DPD is catalyzed by LuxS. (C) The conversion of DPD to AI-2 occurs spontaneously. DPD = 4,5-dihydroxy-2,3-pentanedione.

The LuxS/AI-2 system, found in both gram-negative and gram-positive bacteria, is a regulatory system that allows bacteria to make collective choices about specific gene expression by secreting and identifying a signal molecule.^[43]^ The LuxS/AI-2 system modulates antibiotic resistance in a complex manner. Through its effect on efflux pumps, mobile genetic elements, the VraSR 2-component system, and the folate synthesis pathway, the LuxS/AI-2 system leads to antibiotic resistance.^[52]^ Most importantly, the fact that the LuxS/AI-2 system coordinates QS of bacterial biofilms further contributes to bacterial resistance. The signal molecule AI-2, a furanosyl-borate-diester, is important for signal exchange between different bacterial species.^[53]^ A modified product of 4,5-dihydroxy-2,3-pentanedione (DPD), which is generated and detected by both gram-negative and gram-positive bacteria, has been discovered as a result of the activated methyl cycle.^[53]^ The methyl cycle is shown in Figure 3.

The enzyme S-adenosyl homocysteine hydrolase (SahH) converts S-adenosyl homocysteine (SAH) to homocysteine in a 1-step process, or in a 2-step reaction that requires SAH nucleosidase (Pfs) and LuxS, which catalyze the breakage of the thioether bond of S-ribosylhomocysteine (SRH) to create DPD, which can rearrange to AI-2.^[52]^ The finding that one bacterial species’ AI-2 signal molecule may be recognized by other bacterial species led to the viewpoint that AI-2 is most likely a universal signaling protein involved in inter-species communication.^[52,54]^ LuxS is involved not only in the generation of AI-2 signaling molecules but also in the activation of the methyl cycle in central bacterial metabolism. S-adenosylmethionine functions as a methyl donor in this activated methyl cycle, resulting in the buildup of the toxic intermediate SAH in bacterial cells. The LuxS enzyme helps detoxify SAH with homocysteine and DPD.^[52]^

### 3.3. Other factors

To date, many other factors leading to antibiotic resistance have been identified. Salcedo et al^[55]^ suggested that antibiotics with concentrations lower than their minimum inhibitory concentration can mediate biofilm formation and result in enhanced antibiotic gene transfer. When antibiotics are administered to patients with biofilms, bacterial cells deep within the biofilm are only exposed to very low amounts of antibiotics. Antibiotics have no therapeutic impact in this situation because the low concentration of antibiotics makes them unable to suppress biofilm formation; instead, they operate as inducers, promoting the growth of biofilms.^[56]^ However, the underlying mechanism remains unclear. Another hypothesis is that osmotic stress causes bacterial species to differentiate into a resistant phenotype, resulting in downregulation of transmembrane channels or antimicrobial target sites. Moreover, biofilm persistence may also be revealed by the expression of antimicrobial resistance genes or the use of efflux pumps.^[4]^ Evidence also suggests that fungi and bacteria have synergistic effects on biofilm formation. This was recently supported by evidence from a sheep model that showed that inoculation of the frontal sinus with fungi and *S. aureus* formed more robust biofilms than fungi alone. The exact mechanism is unknown; however, it is postulated that this synergism may be due to the primary pathogenic *S. aureus* establishing a matrix for fungal growth or *S. aureus* causing mucosal injury promoting fungal biofilm.^[15]^

## 4. Discussion

The enormous impact of biofilm-related infections results in large societal expenditures, and related diseases can lead to death in extreme situations. Moreover, the conventional methods have been rendered ineffective owing to the increased multidrug resistance of microorganisms. The EPS generated by biofilms protects bacteria from host immune defense mechanisms and prevents the permeation of antibiotics. Additionally, bacteria within biofilms reduce antibiotic susceptibility through heritable resistance mechanisms, such as adaptive mutations and horizontal gene transfer. The agr and LuxS/AI-2 systems play a key role in antibiotic resistance in CRS. These 2 systems control the expression of a variety of genes and regulate the cellular activities of bacteria to adapt to different environments. Consequently, eradication of biofilms using traditional antibiotics is challenging. There is an essential requirement to explore novel therapeutic strategies that can effectively restrain biofilm-related infections. One strategy is to search for novel antibiotics that can penetrate the interior of biofilms to validate antimicrobial neutralization. Another tactic for biofilm removal is to find an effective surfactant to disrupt the biofilm integrity. Furthermore, because QS regulates the generation of numerous virulence factors and antibiotic resistance genes in bacteria, any activity that interferes with QS signal molecules or receptor-recognizing signal molecules might reduce virulence and inhibit the expression of QS-related genes in bacteria, thereby decreasing the extent of antibiotic resistance in biofilms.

As previously mentioned, signal molecules in QS significantly contribute to antibiotic resistance. We can search for inhibitors of signal molecule synthesis to inhibit QS. The signal molecule AI-2, for example, is vital for signal exchange across bacterial species. For example, the AI-2 precursor S-ribosyl homocysteine is produced by the action of 5-methylthioadenosine (MTAN) on SAH. MTAN inhibition causes accumulation of 5-methylthioadenosine and SAH, as well as a decrease in AI-2 production. AI-2 production is dramatically reduced in MTAN knockout strains and in the presence of MTAN tight-binding inhibitors.^[52]^ Therefore, the application of MTAN to destroy biofilms in CRS is a potential approach. In this way, we can also inhibit signaling molecules and inhibit signal molecule conduction or receptor binding.

Alternative therapies for inhibiting biofilm development in patients with CRS have been proposed because of the limited efficiency of antibiotics. Potential treatment targets that can inhibit and eliminate biofilms formed by CRS-associated bacteria should be developed. However, these therapies have disadvantages. By activating the immune system in sinuses, the use of these chemicals in excessive quantities may cause cytotoxicity and allergic responses. Furthermore, the use of anti-biofilm medicines in the nasal cavity is restricted compared to superficial infections, and the majority of some therapies lack sufficient proof, backed by several well-conducted trials. Although the first in vitro results are promising, further clinical research is needed before these medicines can be widely used. The results of the most resistant CRS cases might be improved by a multimodal therapy paradigm that includes biofilm-specific medications. A new era of CRS management will emerge with our understanding of the intricacy and possible reversibility of CRS biofilms.

## 5. Conclusion

Antibiotic resistance due to bacterial biofilms is a major cause of refractory CRS. By investigating the mechanism of biofilm resistance, we can search for ways to overcome drug resistance and thus provide new ideas for clinical treatment of CRS.

## Author contributions

All authors contributed to the article.

**Conceptualization**: Fengfeng Qin, Lu He, Hui Xia.

**Figures**: Lu He, Sihan Zheng, Jing Liu.

**Resources**: Lanxin Hu, Sihan Zheng, Jing Liu.

**Writing – original draft**: Yanlin Huang, Wenjian Hu.

**Writing – review & editing**: Ji Yin, Sen Li.
